# Newborns' temperature submitted to radiant heat and to the Top Maternal device at birth

**DOI:** 10.1590/1518-8345.0305.2741

**Published:** 2016-08-08

**Authors:** Rosemeire Sartori de Albuquerque, Corintio Mariani, Ana Aparecida Sanches Bersusa, Vanessa Macedo Dias, Maria Izabel Mota da Silva

**Affiliations:** 1 PhD, Professor, Escola de Artes, Ciências e Humanidades, Universidade de São Paulo, São Paulo, SP, Brazil.; 2 PhD, Professor, Universidade Cidade de São Paulo, São Paulo, SP, Brasil. Technical director, Hospital Maternidade Leonor Mendes de Barros, São Paulo, SP, Brazil.; 3 MSc, Researcher, Hospital Maternidade Leonor Mendes de Barros, São Paulo, SP, Brazil.; 4 RN, Midwife, Hospital Maternidade Leonor Mendes de Barros, São Paulo, SP, Brazil.

**Keywords:** Body Temperature Regulation, Newborn, Delivery, Mother-Child Relations

## Abstract

**Objective:**

to compare the axillar temperatures of newborns that are put immediately after birth in skin-to-skin contact under the Top Maternal device, as compared to those in a radiant heat crib.

**Methods:**

comparatives observational study of the case-control type about temperature of 60 babies born at the Obstetric Center and Normal Delivery Center of a public hospital of the municipality of Sao Paulo, being them: 29 receiving assistance in heated crib and 31 in skin-to skin contact, shielded by a cotton tissue placed on mother's thorax, called Top Maternal.

**Results:**

the temperature of the babies of the skin-to-skin contact group presented higher values in a larger share of the time measures verified, as compared to those that were placed in radiant heat crib, independently from the place of birth. Differences between the two groups were not statistically significant.

**Conclusion:**

the study contributes to generate new knowledge, supporting the idea of keeping babies with their mothers immediately after birth protected with the Maternal Top, without harming their wellbeing, as it keeps the axillar temperature in recommendable levels.

## Introduction

Giving birth was historically considered natural and physiological, differently from how is seen nowadays. In the first decades of the 20^th^ century and along the industrialization of Brazil, it became to be perceived as a pathological process demanding control with the aim of avoiding maternal and perinatal mortality^1^.

After the Second World War, and following the medical improvement in delivery care, pregnant women began to be hospitalized to have their babies. It thus began the predominance of surgical interventions in a process until then considered normal and belonging to the household by the society^1^. It is subject of general understanding that maternal and perinatal mortality have been experiencing slow but continuous decrease year after year. Regarding the care of the newborn, technologies have improved prognosis, although the hospital-centered model that was imposed provoked an alienation of mother and baby immediately after delivery. There is a perception that these hospital routines may interfere with the early interaction between mother and child, with harmful effects on both^2^.

Several common procedures used in delivery care, birth and early post-partum, impact on the transition from fetus to newborn, including medication used during delivery, aspiration protocols, strategies for avoiding heat loss, clamping of umbilical cord and using 100% oxygen for reanimation^3^ ^-^ ^4^.

A review of the scientific evidence about the commonly used procedures in newborn care allows the conclusion that many of these procedures previously cited have no proved efficacy and pointed to changes or amendments due to the fact that they interfere negatively in mother-baby relationship. Regarding cord clamping, and following the available evidence, it should be delayed, as a way of preventing infant anemia. Other studies show no benefit in routine aspiration of mouth and nose at birth. Gastric aspiration shows to be harmful, and it needs to be better prescribed and not used routinely. Even resuscitation maneuver is recommended to be done with atmospheric air firstly, waiting to use 100% oxygen only when there is no success in this first step^3^.

Regarding putting the newborn naked, wiped dry, in prone position on the mother's chest immediately after birth, called skin-to-skin contact (STSC) or immediate contact, several benefits are highlighted: increased breastfeeding, more stable cardiac frequency, cry after birth and weight stabilization, added to a reduced chance and lower time of ICU hospitalizations and efficiency in thermo-regulation of the newborn, that will be the focus of the present study^3^.

As a consequence of the previous considerations, it is expected that through the implementation of the skin-to-skin contact practice as a substitute of the immediate interventionist separation, all the benefits of this model will be achieved, and the newborn temperature will stay in the desired level of thermo-regulation.

After having experience in caring during the first hours of life and evidences on the newborn been in skin-to-skin contact, this study had as its objective to compare the axillar temperature of newborns that had skin-to-skin contact immediately after birth, with the aid of the Top Maternal device, as compared to the temperature of those placed in radiant heat crib.

## Methods

This is a comparative observational study, of the case-control type about two different ways of care to keep thermo-regulation in newborns after delivery. In one of them, the newborn immediately after birth, is wiped dry, naked (just diapers), and is placed on a heated source (heated crib) and received the care as needed in this site (apart form the mother). In the other, baby was also wiped dry and without clothes, only diapers, is placed on the mother's thorax and under a device developed by the Maternity Hospital Leonor Mendes de Barros (HMLMB), branded Top Maternal, and received care jointly with the mother.

The Top Maternal device has been in use for five years in the HMLMB, and it is made of cotton tissue in a circular shape, 90 cm wide and 90 cm height, and placed immediately before delivery in the mother's thorax covering the breast. It was conceived with the pre-supposition of attending two key requests of mothers that were cared by in the Hospital and also by the nursing team: the first was related to protecting the bosom during delivery and reducing the embarrassment related to body exposure, and the second was to help in the safety of the team regarding the potential slipping and fall of the baby, as it is a placed in contact with the skin and is thus been held between the tissue and the maternal chest.

The present research was performed in the wards of the HMLMB called "Center for Normal Delivery -CPN" that are mainly attended by Obstetric Nurses and "Obstetric Center - CO" that is predominantly attended by Obstetricians (physicians). The hospital is the reference unit for maternal-fetal high risk in the Southeast region of the municipality of Sao Paulo, having an expressively high number of deliveries. Data from the national health information system for the Brazilian health system (DATASUS) show that 4194 deliveries happened there in 2013. It is worth noting that the hospital has allowed unrestricted access to risk pregnancies, and usually cares for foreigners that live in the downtown area of Sao Paulo, frequently rejected in other maternities. It is usual to give care to women from other countries, and especially from Latin America, the Bolivians. 

Newborn reception, both in CPN and CO is performed by Neonatologist physicians that determine at birth, and depending on the conditions of the baby at that moment, the site where care will be given, if in skin-to-skin contact or in radiant heat crib.

For obtaining more precision in the results analysis of body temperature of newborns in CO and CPN, environmental temperatures in both sites were recorded. In the CO the average temperature was 24^o^C, and kept by means of central control air conditioning, keeping this value stable as per the thermometer in the delivery room. In the CPN, temperature is atmospheric, not having air conditioners and is recorded by a thermometer in each delivery room, and during the same period it was kept around an average of 23^o^C. 

The population under study was made of 60 women in labor and their newborns. Data collection happened between February 2013 and March 2014, in the 8 am-3 pm shift, Monday to Friday, the time when the team with training for data collection was present. It was used as a basis the studies with the same research objectives (skin-to-skin contact) such as the one in Australia in 2014 that showed benefits in regard with breastfeeding^5^.

The present study was approved by the Committee for Ethics in Research of the HMLMB under verdict number 176.708.

The inclusion criteria of the women in labor were: age equal or higher than 18 years, gestational age between the 37^th^ and 41^st^ week plus one day, to ensure the physiological conditions of newborns; mother's consent in using the tissue device during delivery to allow to place the newborns immediately under it, accepting to participate in the study, authorizing to measure the axillar temperature of their babies in three moments: fifth, tenth and thirteenth minute after birth. The record of these three values was because it was needed to know if, in contact with the skin, there is sustained and preserved babies temperature, an effect that is expected in the first minutes after birth^3^.

In practice, the largest share of maternities remove the babies from their mothers' contact before this recommended period, precisely in the early moments after birth, to perform some routine procedures under radiant heat, allegedly for protecting the baby from heat loss. There were also considered for inclusion in the study the newborns that had Apgar scores >7 in the first and fifth minute of life, after Neonatologist's assessment. This measure gives reassurance that persistent hypothermia cases will be identified and urgent intervention will be performed.

Newborns from mothers with complications at delivery, with fetal distress and those that needed neonatal reanimation, were excluded.

The data collection form was designed for the transcription of the information about the profile of the woman in labor, as per data coming from the clinical records (Age, gestational age, self-referred race, and nationality) and from the newborn clinical record (Birth conditions as per Apgar Score in first and fifth minute), weight at birth. At this point of time, the axillar temperature values (that are not part of routine hospital measures) at the fifth, tenth and thirstiest minute of life were recorded through a researcher trained for this measuring, using a digital thermometer that was specific for the study. The technique was to place the instrument in the newborn's axilla until the alarm set off (average two minutes) in a determined timing, and then values were recorded in the newborn's clinical sheet and transcript to the research form.

The study observed the national and international norms of ethics in research involving human beings.

## Results 

Related to the 60 mothers' profile included in the study, data regarding age, gestational age in weeks, race and nationality were included. Comparing the profiles of the mothers whose newborns were sent to radiant heat or to skin-to-skin contact, there was a fairly equal distribution among the study population. 

The average age of the sample was 39,07 years old, in the range from 31 to 41 years, and the more prevalent age was 39 years (33,3%). Regarding skin color, white was predominant as well as the Brazilian nationality. Gestational age, even considered full-term from 37 to 42 weeks^6^ as well as in the majority of studies on labor, that show the beginning of labor around the 38^th^ week, both women pertaining to Skin-to-Skin Contact Group (CG) and from the Radiant Heat Group (RHG) had hospitalization, labor and delivery in a large proportion (47%) with gestational ages between 37 and 38 weeks.

**Figure 1:**
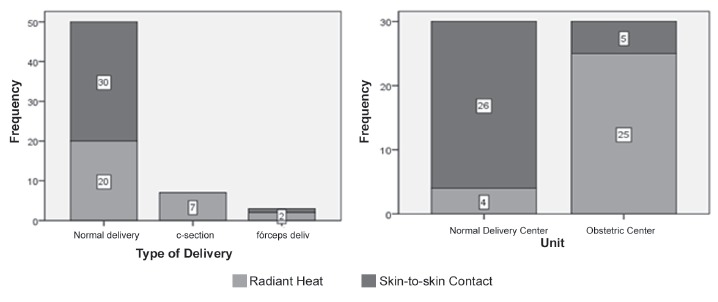
Type of intervention (Skin-to-skin contact under Top Maternal and Radiant heat) per type and site of delivery of newborns at the Hospital Maternity Leonor Mendes de Barros. São Paulo, SP, Brazil, 2014

Regarding the different types of delivery and the site where they happened, Figure 1 shows that normal delivery was more frequent in the majority of cases in both groups (83,3%), C-sections with 11,7% were in a second place, and lastly came forceps delivery with 5,0 %. The distribution between radiant heat crib and skin-to-skin contact showed that the normal deliveries were mainly sent to the CG and those born through C-section were 100% sent to radiant heat crib. 

Data collection happened in CPN and CO. Checking the distribution of referrals of newborns to radiant heat cribs or skin-to-skin contact comparative groups, it was noted that in CPN the referral to skin-to-skin is 93,3% while in the births in the CO the proportion of this behavior is the inverse (16,6%).

Regarding the conditions at birth as per the Apgar score of the 60 babies under study, the majority had a score of nine in the first minute and ten in the fifth minute, configuring a good condition in all the studied babies. 

**Figure 2:**
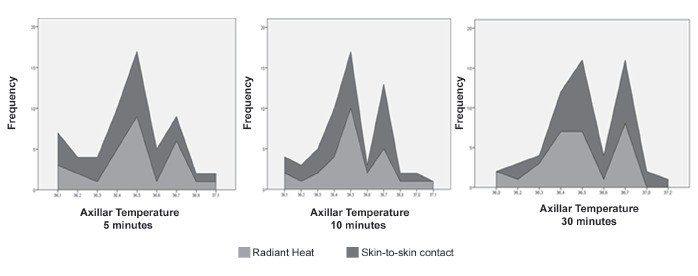
Graphic representation of the trends in axillar temperature of newborns in the 5th, 10th and 30th minutes of life sent to radiant heat crib and placed in skin-to-skin contact with Top Maternal device. São Paulo, SP, Brazil, 2014.

Weight in newborns ranged from 2.100Kg to 4.210Kg with an average of 2.984Kg. The majority (55%) had a weight between 2.780Kg and 3.500Kg (Figure 2).

Figure 2 and Table 1 show data from main outcomes of the study, regarding the comparison between the values of temperatures of newborns in the 5th., 10th. and 30th. minutes of life, sent to radiant heat cribs and the temperature values for the same three times for the newborns placed in skin-to-skin contact using the Top Maternal device, taking into consideration the two delivery sites temperatures ranged between 23^0^C e 26^0^C.

**Table 1:** Axillar temperature measures of newborns in the 5th., 10th. and 30th. minutes of life, sent to radiant heat cribs (RHG) and the temperature values for the same three times for the newborns placed in skin-to-skin contact using the Top Maternal device (CG) and site of delivery. São Paulo, SP, Brazil, 2014.

Groups	Delivery site	Minutes of life	Average	Standard Deviation	Minimum	Median	Maximum	N
RHG^*^ Obstetric Center (CO) 5			36,52^†^	0,23	36,1	36,5	37,1	25
		10	36,52^‡^	0,24	36,1	36,5	37,1	25
		30	36,47^§^	0,20	36,0	36,5	36,7	25
	Normal	5	36,33^†^	0,10	36,2	36,4	36,4	4
	Delivery	10	36,53^‡^	0,15	36,4	36,5	36,7	4
	Center	30	36,48^§^	0,17	36,3	36,5	36,7	4
	Obstetric	5	36,44^†^	0,22	36,1	36,5	36,7	5
CG^||^	Center	10	36,54^‡^	0,17	36,3	36,5	36,7	5
		30	36,54^§^	0,15	36,4	36,5	36,7	5
	Normal	5	36,46^†^	0,23	36,1	36,5	37,1	26
	Delivery	10	36,49^‡^	0,22	36,1	36,5	37,0	26
	Center	30	36,58^§^	0,23	36,2	36,5	37,2	26

*RHG Radiant Heat Group. ||CG Skin-to-Skin Contact Group

The descriptive level of a test (also known as p-value) express the probability of making an error when rejecting the hypothesis, while it actually being true. In the largest part of tests, the equality hypothesis, in the case of Table 1 is that the average of the groups are all the same: † p-value = 0,5547; ‡p-value = 0,6305; §p-value = 0,0556.

The axillar temperature of newborns placed in contact skin-to-skin under the Top Maternal, both for those born in the CO and for those born in the CPN, had a value a little higher when compared with the temperature of those newborns placed in radiant heat crib. Specifically in the CO, in the third and thirstiest minute the temperatures were 36,46^0^C and 36,58^0^C respectively. The ANOVA (Analysis of Variance) test showed that there were no periods when differences were significant with p-value=0,5547 in five minutes, p-value=0,6305 in ten minutes and p-value=0,0556 in 30 minutes.

## Discussion

The analyses presented in this study were comparative between two groups: one considering that newborns soon after birth were exposed to radiant heat (RHG) and the other were placed in skin-to-skin contact (CG). In both groups, the age of the mothers ranged from 31 to 41 years, with the majority (56.6%) aged 39 to 40 years Predominance of white color skin in 68.3% and gestational age between 37 and 38 weeks 45.0%, not differing from the general profile seen in HMLMB. Brazilian mothers prevailed with 75.0%, and foreign, in the case Bolivians, accounted for 16.7%.

Regarding the profile of the newborns included in the study, the results showed that the value of the Apgar score for the first minute (66.7%) was nine for the majority, and of these, (67.5%) were represented in CG. In the fifth minute, all (100.0%) infants had Apgar score above nine, with 85.0% in the value of 10, also represented the majority (56.8%) were in those in the skin contact group. The Apgar score is a clinical tool to help identify newborns who require resuscitation, and in it, are considered indicative values > 7 and <7 for good or bad condition of birth respectively^7^. Thus, it is clear that all newborns included in the study were born in good conditions, and also kept in those conditions in the fifth minute of life, since at this time, all (100.0%) had scores between 9 and 10.

There were no significant differences in the profile of mothers and babies between the two arms of this study.

The main objective of this study was to find out if there was a significant difference between the temperature of newborns that received care away from her mother at birth in radiant heat crib, a practice commonly used in the management of reception of newborns in the delivery room, vs. the temperature of those who were put together their mothers in skin-to-skin contact favored by the Top Maternal device.

The newborns' temperature stability occurs when there is a balance between production and removal of heat. Therefore, every effort should be made to avoid heat loss immediately at birth.

At birth, the baby passes from the intrauterine milieu where the proper temperature for its wellbeing is maintained at around 37.5 ° C, for an extra-uterine environment, the cooler and dryer environment of the delivery room, fostering heat loss by evaporation and convection. To avoid loss of temperature, there is a need for attention from the team that receives the newborn immediately after birth, which conventionally include: wiping dry the baby, putting it in radiant heat source with radiant crib and monitor axillary temperature in a constant or continuous way.

Many of the practices used routinely have not been proven effective and therefore they should be modified as they interfere negatively, such as not to maintain the baby with its mother soon after birth. The practice of placing the newborn at the moment of birth in skin to skin contact with his mother, is a safe procedure without great cost and suitable for regulating body temperature of healthy newborn, being recommended by the Clinical Practice Guideline of Normal Delivery Care (2010)^8^ and by the NICE Directive (2014)^9^. 

Although there is a clear recommendation and recognition through evidences, regarding the early contact between mother and newborn, this practice, according to studies on the subject, seems not to receive due attention from those health professionals responsible for performing the vast majority of deliveries and births nowadays, since most of the babies are still placed in the first hours of life under radiant heat^10^.

This practice is also strengthened by the fourth step of the Ten Steps to Successful Breastfeeding^11^, which helps women to start breastfeeding in the first hour after birth and, to this end, recommends early and lasting skin-to-skin contact in the immediate postpartum period, which should last until the first breastfeeding or as long as the mother desires. This early or immediate contact, as evidenced in this step means putting the baby naked in the prone position on the mother's chest immediately after birth. 

In this sense, some steps have been observed and described by sequencing the first actions of the newborn, when placed in skin to skin contact with the mother, ranging from crying to the recognition of the nipple (through smell), the breastfeeding and sleep. The evidence with respect to temperature are significant; when there is neutrality between intra-uterine and extra-uterine life, it gives an opportunity to the newborn to have a better match to the physiological patterns of oxygen saturation and heart rate. Sensorial stimuli such as touch, heat and odor, are vagal exciters that, among other effects, release the maternal oxytocin, which works by increasing the mother's breast skin temperature, providing warmth for baby^3^.

If the baby has good vitality at birth, in addition to the normal procedures, heat should be provided in a way that body temperature may remain between 36.5 ° C to 37.0 ° C.

In this study, no temperatures lower than 36,0^0^C were recorded, even 30 minutes after birth, in which 65% of newborns were within the normal range, between 36.5 to 37 ° C and just 35% showed mild hypothermia between 36.0 and 36.4 ° C. However, in the CG, only 38.9% developed mild hypothermia after 30 minutes, while the RHG reached 61.9%, leading to the conclusion that putting the baby in skin to skin contact or together with its mother collaborates with maintaining its body temperature, as compared to those placed in the radiant heat and away from its mother, regardless of place of birth or type of delivery.

A meta-analysis comprised by 23 studies indicated strong evidence of increased body temperature in skin to skin contact, and a remarkable fact is that the ambient temperature does not influence the outcome body temperature, as even in colder environments, the body temperature of newborns in skin-to-skin contact had increases or at least remained unchanged^3^.

The data in this study showed similar results to those described above, and also in other studies^12^ ^-^ ^14^, identifying that the room temperature where the newborns were left after birth had scarce influence in maintaining their body temperature, as shown in Table 1. The room temperatures ranged from 23^0^C to 26^0^C and most (56.6%) of births happened with temperatures at 23^0^C. In the CG, 51.6% kept their body temperature after 30 minutes from 36,4^0^C to 37,2^0^C, while in the RHG, in 48.4% the temperature ranged from 36,0^0^C and 36,7^0^C, leading to the conclusion that the skin contact kept the newborns under study with temperatures near the normal range, while for those who were in radiant heat, the values ​​were closer to the mild hypothermia range.

It is well known that the type of delivery may influence the newborn's temperature shortly after birth, especially when after C-section and when the mother have received spinal anesthesia. In anesthetic process, there is a reduction of the mother's body temperature, leading also to a decrease in the baby's temperature^3^. Nonetheless, in this study, only 28.0% in the caesarean delivery newborns belonging to the RHG had temperatures recorded at 36,0^0^C, and only after 30 minutes of birth. Noteworthy is the fact that in normal deliveries in the CPN, the vast majority (86.6%) of the babies were placed in skin to skin contact, inversely proportional to what was observed in the CO deliveries, where 20 (66.6%) were normal, seven (23.3%) C-sections, and three (10.0%) were by forceps. Most (83.3%) were referred to radiant heat, reinforcing the idea that even in babies in good condition, there is a greater tendency for a model of care in which the majority of care is given by nurses than for the model where newborns are assisted in skin to skin contact with their mother.

Limitations of this study are related to the difference in the temperature control of the two environments involved, one with air conditioning and another at room temperature, and also to the number of the population under study. Although the temperature values ​​were similar, it is suggested in the occasion of another study, to include larger number of subjects and birth environments with the same temperature, so that this variable is controlled.

## Conclusion 

In this study, it was shown that the temperature of the newborns that were placed in skin-to-skin contact with the aid of Top Maternal device soon after birth, showed higher values in most checked times, compared to the temperature of those placed in a heated crib under radiant heat, regardless of place of birth, and that there were at no time, temperature values ​​suggestive of moderate or severe hypothermia. When subjected the data to statistical analysis, it was found that there was no significant difference between groups.

The findings corroborate the latest evidence in the literature on the temperature of newborns soon after birth, and present the results in a tropical country whose climatic characteristics are different from those of countries that developed most studies included in this research. It also brought to light the assumption that infants that are not subject of radiant heat crib care shortly after birth can experience diminished temperature compromising its balance, making this an unnecessary concern. Furthermore, this study contributes to the advance of knowledge that supports keeping babies with their mothers immediately at birth, favored by skin contact under the use of Top Maternal, without prejudice to their well-being, since the temperature of newborns on average remained at recommended levels. Also favored the benefits that skin-to-skin contact brings to both the mother and the newborn and also for the management of services that should rethink the routine use of radiant heat equipment.
